# Efficacy dilution in randomized placebo-controlled vaginal microbicide trials

**DOI:** 10.1186/1742-7622-6-5

**Published:** 2009-10-09

**Authors:** Benoît R Mâsse, Marie-Claude Boily, Dobromir Dimitrov, Kamal Desai

**Affiliations:** 1 Statistical Center for HIV/AIDS Research & Prevention, Fred Hutchinson Cancer Research Center, Seattle, Washington, USA; 2 Department of Infectious Disease Epidemiology, Imperial College, UK

## Abstract

**Background:**

To date different vaginal gel microbicides have been evaluated in phase 2b/3 trials, but none have demonstrated effectiveness for preventing HIV infection. Failure to demonstrate effectiveness however does not necessarily indicate that a product is truly inefficacious, as several sources of efficacy dilution may compromise our ability to identify products that may have been truly efficacious.

**Methods:**

For four individual sources of dilution, we describe the dilution mechanisms and quantify the expected effectiveness. An overall expected effectiveness that combines all sources of dilution in a trial is derived as well.

**Results:**

Under conditions that have been observed in recent microbicide trials, the overall expected effectiveness assuming an active gel with true efficacy of 50% and 75% are in the range of [16%; 33%] and [28%; 50%], respectively, when considering the four major sources of dilution. In contrast the diluting effect due to adherence alone (assuming an adherence of 80%) leads to higher expected effectiveness, 40% and 60% assuming an active gel with true efficacy of 50% and 75%, respectively. Individual sources of dilution may demonstrate a small effect when evaluated independently, but the overall dilution effect in a trial with several sources of dilution can be quite substantial.

**Conclusion:**

Currently planned phase 2b/3 microbicide trials of new candidate vaginal microbicides are not immune from these shortcomings. A good understanding of dilution effects is necessary to properly interpret microbicide trial results and to identify products worthy of further development and evaluation. Greater attention should be devoted to reducing and assessing the impact of efficacy dilution and to carefully selecting the effect size in the design of future trials.

## Introduction

The development of female initiated HIV prevention methods is a high priority. For this reason, several different vaginal gel microbicides have been evaluated in phase 2b/3 efficacy trials, but to date none have demonstrated effectiveness while several have suggested evidence of harm. Table S1 reviews the findings of phase 2b/3 microbicide trials completed to date (see Table S1 in additional file 1). For instance, the cellulose sulfate gel (Ushercell, Polydex Pharmaceuticals, Toronto, ON, Canada and Topical Prevention of Conception and Disease [TOPCAD], Chicago, IL, USA) was found to be ineffective in preventing HIV infection and may also have potentially increased the risk of HIV acquisition in women [1]. The candidate microbicide Carraguard (Carraguard/R515, Population Council, New York, NY, USA) was found to be safe for vaginal use but ineffective in preventing male-to-female HIV transmission in a phase 3 trial [2]. More recently the HPTN-035 trial of 0.5% PRO2000 gel (Endo Pharmaceuticals, Chadds Ford, PA, USA), a viral entry inhibitor, reported an estimated effectiveness of 30% (95% CI [-8%; 54%]) which, although of borderline significance (p = 0.10), was welcomed with optimism [3]. Results of the MDP-301 study, a second phase 3 trial of 0.5% PRO2000, are expected at the end of 2009, and results of other candidates are also forthcoming [4].

The outcomes of these microbicide trials deserve a closer look as failure to demonstrate the effectiveness of a product in a phase 2b/3 trial does not necessarily imply that the product is truly inefficacious. Different "diluting" factors present in all trials (see Table S1) could compromise our ability to detect the true efficacy of a microbicide. Failure to clearly understand the potential impact of the dilution factors in microbicide trials could lead to misinterpretation of trial results and the discarding of efficacious products from further evaluation. Thus, it is important to quantify the relationship between the diluting factors and the expected reduction in true efficacy in microbicide trials. The objective of this paper is to assess the expected magnitude of the efficacy dilution in microbicide trials resulting from the most frequent sources of dilution, namely product adherence, time off-product due to pregnancy, HIV infections from anal intercourse and the inertness of placebo gel. This paper extends the works of Desai [5], Desai et al [6] and Weiss et al [7] which focus only on adherence as one potential dilution factor in HIV prevention trials.

## Methods

### True Efficacy, Expected and Observed Effectiveness

In order to formulate the relationship between the dilution factors and effectiveness, we first define our use of the term efficacy, expected and observed effectiveness. We define the true efficacy, *E*_*T*_, as the efficacy of a product used under optimal trial conditions when compared to a completely inert product. The term observed effectiveness refers to the intention-to-treat (ITT) estimate obtained from a trial, i.e. one minus the ratio of the observed HIV incidence rate in the active gel arm over the incidence in the placebo arm. The term expected effectiveness is the expected value of the observed effectiveness (i.e. without random variability associated with the conduct of a trial). In absence of any source of bias or dilution, the expected effectiveness should be equal to the true efficacy. In presence of dilutions, the expected effectiveness will be less than the true efficacy. Thus, the dilution effects are expressed in terms of *E*_*T*_, the true efficacy of a candidate microbicide (active gel). We describe in Table S2 the details of the dilution mechanisms of the true efficacy and the resulting expected effectiveness for each factor acting alone (*E*_*a*_, *E*_*o*_, *E*_*s*_, and *E*_*b*_) and in combination *E*_*all *_(see Table S2 in additional file 2). Without loss of generalisability, we restrict our analysis to the two arm randomized trial using a 1:1 allocation. Homogeneity assumptions are made for simplicity. Although these assumptions might appear unrealistic, the introduction of more heterogeneities can in some cases amplify the impact of the dilution.

### Product Adherence

A proportion, *a*, of women (adherents) in the trial are assumed to use the microbicide as prescribed by the protocol for each vaginal intercourse during the entire follow-up. This definition implies that non-adherent women never use the gel during the trial therefore; they do not get any benefit from the gel as gel is never used. A more realistic alternative model might be preferable where women may use the gel for some acts of vaginal intercourse and may not for others [7,8]. Under this alternative model, women using the gel for some but not all of the vaginal acts would potentially benefit less than those that are always using the gel. However, the dilution effect under this alternative model is quite similar to that where women are either classified as adherent or not adherent.

Product adherence estimates from completed microbicide trials vary greatly (see Table S1). The high variability across trials can be partly attributed to different methods of assessment. Self-reported adherence from face-to-face interviews tends to over-estimate product adherence because of social desirability bias [5]. Therefore, most estimates of adherence in Table S1 over-estimate the true proportion of adherent women.

### Time Off-Product Due to Pregnancy and Adverse Reactions

All candidate vaginal gel microbicides under development are investigational products without human data on their safety in pregnancy and to the fetus/embryo. Although, the product's active ingredients of most candidates are not absorbed by the genital mucosa, the fetus/embryo may still be exposed due to the passage of the product through the uterus and the cervical canal [4]. Given that harm from the product cannot be entirely ruled out, microbicide protocols have cautiously address the issue (1) by excluding women who have the intention of becoming pregnant during the trial or requiring women to use at least one or two contraceptive methods and (2) by temporarily withdrawing the product from women becoming pregnant during the trial with product use resuming after pregnancy [9]. Despite these precautions, pregnancies occurring during follow-up have lead to high proportion of follow-up without product use in many trials. Pregnancy rates between 16 and 64 per 100 woman-years have been observed in microbicide trials which can lead to a proportion of follow-up time off-product between 5% and 20% [9]. Although a product can also be withdrawn from women for other reasons such as the occurrence of adverse reactions, the dilution effect is negligible because the time off-product due to other emerging conditions is substantially smaller than the one due to pregnancy. Thus, we assume that a proportion, *o*, of total follow-up time will be off product due to pregnancy.

### Anal Intercourse (AI) as a Source of HIV Infection

Most candidate microbicides currently under development aim to prevent transmission from vaginal intercourse such that most protocols prohibit the use of these products for AI as well as counseling participants to abstain from unprotected receptive AI. Thus, we assume that a proportion, *s*, of HIV infection endpoints was acquired from unprotected receptive AI. Due to the sensitive nature of this practice, the prevalence of unprotected AI has been notoriously hard to estimate and is believed to be greatly under-estimated both in trials and in behavioral surveys. A NSFG 2002 survey has estimated that 34% of US women reported having AI with an opposite sex partner in their lifetime [10]. In contrast, among sexually experienced South African youth, 5.3% of women reported ever engaging in AI [11]. In completed trials, the reported rate of ever having AI varies between 14% in a lifetime to about 2% in the past month (see Table S1). Such large differences are due partly to variation across clinical sites and to the data collection methods used to capture sensitive behavior. Therefore, these trial estimates of AI are most likely under-estimating the true frequency of AI.

### Placebo Physical Barrier/Lubrication Effect

Up to five different placebo gels have been used in microbicide trials, many of which may not be completely inert [4]. For instance, the methycellulose gel used as the placebo in the Carraguard trial may have acted as a physical barrier and reduced HIV acquisition [12,13]. Furthermore, given the potential role of the vaginal pH on transmissibility of HIV, it is somewhat problematic that active gel and placebo have different pH levels affecting the vaginal flora in presence of semen. For example, the placebo gel used in the cellulose sulfate trials has a pH level of 4.4 compared to a pH level of 7.5 for the cellulose sulfate gel. If truly effective in reducing HIV acquisition, the efficacies of the placebo gels currently used in microbicide trials are believed to be relatively small (*E*_*p*_< 10%). For instance, the HPTN-035 trial observed an hazard risk ratio of 0.97 (95% CI [0.66;1.44]) when comparing the placebo gel to the 'no gel' arm (although condom use was significantly higher in the 'no gel' arm).

### Total Dilution Effect and Overall Expected Effectiveness

In a given microbicide trial, some or all of the above dilution factors will be present. If we assume a weak or no association between pregnancy risk, adherence, and frequency of unprotected receptive AI, the overall expected effectiveness *E*_*all *_can be derived using the natural ordering described in Table S2. However, if women engaging in unprotected AI were also lower gel users (i.e. non-adherent) and had higher pregnancy rates, then the dilution effects would be concentrated among a relatively small proportion of participants. This clustering would lead to a total dilution effect that would be smaller than the one obtained under our weakly dependent model. However, we argue that this strong clustering is unlikely since if dilution factors were highly correlated this would also mean that most trial participants would be highly adherent, not engage in unprotected receptive AI, and have very low rates of pregnancy; an association that has not been observed in trials. The total expected efficacy dilution (*D*) is simply the percent reduction between the true efficacy of the product *E*_*T *_and the expected effectiveness in a randomized controlled trial (*E*_*all*_).

## Results

**Figure 1:**
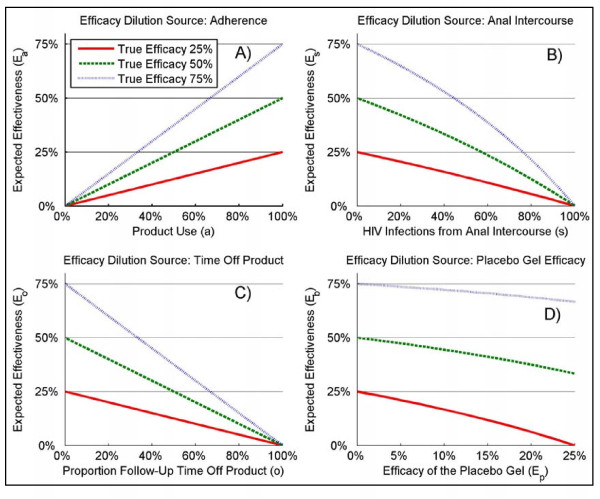
**Expected effectiveness for different source of dilution**. For different level of true efficacy of a candidate microbicide; *E*_*T *_= 25%, 50%, and 75%; Panel **A **shows the results for the dilution due to adherence (*E*_*a*_), Panel **B **shows the results for the dilution due to HIV infections from anal intercourse (*E*_*s*_), Panel **C **shows the results for the dilution from time off-product due to pregnancy (*E*_*o*_), and Panel **D **shows the results for the dilution due to the efficacy of the placebo gel (*E*_*b*_).

The target adherence when designing microbicide trials has often been set to achieve at least '80%', which is higher than the observed adherence depending on the assessment methods (see Table S1). For this level of adherence the expected effectiveness *E*_*a *_is 40.0% when the true efficacy of the active gel is 50% (Figure 1A). Similarly, 20% of follow-up time off-product due to pregnancy yields an expected effectiveness *E*_*o *_of 40.0% (Figure 1C). In a trial where 15% of the observed HIV endpoints come from unprotected AI, the expected effectiveness *E*_*s *_would be 44.2% (Figure 1B). Finally, if the placebo gel has an efficacy *E*_*p *_of 10%, the expected effectiveness *E*_*b *_is 44.4% (Figure 1D). Therefore, using these values for the dilution parameters, the percent reduction in efficacy *D *for an active gel with a 50% true efficacy ranges between 11.2% and 20.0%. The corresponding dilution ranges for an active gel with true efficacy of 25% and 75% are [13.3%-33.3%] and [3.7%-20.0%], respectively. The expected dilution will be more pronounced for smaller true efficacies *E*_*T *_for the dilution effect due to placebo efficacy and AI. For the latter the difference is not substantial, but for the former the dilution effect will be more substantial, especially when the true efficacy *E*_*T *_is relatively close to the efficacy of the placebo *E*_*p*_.

**Figure 2:**
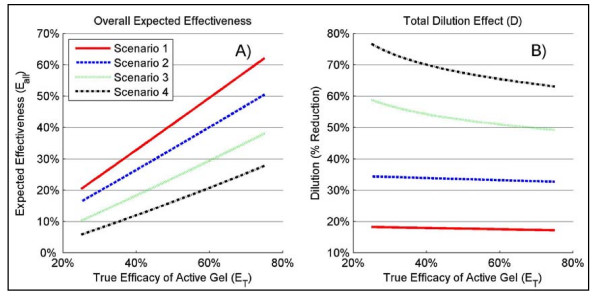
**Overall expected effectiveness and total dilution effect**. For the four different scenarios described in Table S3 (see Table S3 in additional file 3) and as a function of the true efficacy of a candidate microbicide (*E*_*T*_), Panel **A **shows the results for the overall expected effectiveness (*E*_*all*_) and Panel **B **shows the results for the total dilution effect (*D*).

Using different values for the dilution parameters, we have constructed four scenarios to investigate the total dilution effect when all four sources of dilution are taken into account (see Table S3 in additional file 3). Scenario 1 represents the somewhat ideal microbicide trial with very high adherence, low pregnancy, small proportion of HIV infections from AI, and no placebo efficacy. Scenarios 2 and 3 reflect data from completed microbicide trials (Table S1) and from field studies (frequency of AI), although estimates for adherence and AI obtained from trials are likely over- and underestimated, respectively [7,9,11]. We have set the placebo efficacy at 0% and 5% for these scenarios since there are no data available for many of the different placebo gels that we used in the completed trials. Scenario 4 represents an extreme but not unrealistic example of a trial with strong dilution effects from all sources. The results for the total dilution effect *D *and the overall expected effectiveness *E*_*all *_are also presented in Figure 2. For the most optimistic scenarios 1 and 2, the total dilution effects are in the range of [17.2%-18.3%] and [32.7%-34.4%], respectively, while for scenarios 3 and 4, *D *ranges from [49.2%-58.8%] and [63.0%-76.6%], respectively.

Assuming that the true efficacy of an active gel is 50% under scenarios 3 and 4, the overall expected effectiveness *E*_*all *_are 33.2% and 23.8%, respectively, a range below what trials typically have been powered to detect. Under the more extreme scenario 4, the overall expected effectiveness is only 16.3% while it is 41.3% under the ideal scenario 1. Importantly, although the latter appears small, the effect is substantial when put in terms of statistical power. For instance, 66 HIV endpoints are required to achieve 80% power with a 2.5% false positive error rate in a 2-arm trial in order to detect a 50% effectiveness while an additional 45 HIV endpoints (for a total of 111) are required to detect an effectiveness of 41.3% instead of 50%. An increase of 45 HIV endpoints would require a substantial amount of additional resources.

## Discussion

Effectiveness trials for vaginal gel microbicides have several sources of efficacy dilution, most or all of which are present in trials that have been completed so far. We have illustrated that when all these sources of dilution are present within the same trial, a substantial dilution of the true microbicide efficacy may ensue. Under the levels of dilution reported from completed microbicide trials the overall expected effectiveness will be in the range of 24% and 33% when the true efficacy of active gel is 50%. Under more severe dilution conditions, the expected effectiveness could be as low as 16%.

Our analysis has several limitations. Our model is simple and relies on an assumption of weak association between the different dilution factors. Although more complex models could be developed, it would require several assumptions on the relationships between the different sources of dilution. Those additional assumptions could be justified on the basis of the associations observed in microbicide trials. Nevertheless, our findings on the dilution effect due to adherence alone replicate those by Weiss et al [7] obtained using a different model. For 80% adherence and true efficacies of 25%, 50%, and 75%, they have obtained expected effectiveness of 18%, 37%, and 57%, respectively, compared to 20%, 40%, and 60% under our model. Therefore, the use of the Weiss et al model for the dilution due to low adherence would lead to slightly larger dilution effect. Our definition of adherence includes women who are using the gel inside the appropriate time interval before intercourse as prescribed by the protocol (e.g., within two hours prior to intercourse) and assumes that the efficacy of a product used outside the time interval would be greatly diminished or even completely eliminated. To our knowledge, no reliable data is available on gel applications that are outside the time interval, but further dilution can occur if gel users are not using the product within the correct time interval. As well, we have not considered other potential dilution factors such as product sharing among trial participants who are assigned to different arms of the trial. Only anecdotal evidence has been reported so far, and data on product sharing remains scarce or even inexistent. In addition, further dilution of efficacy can result from a strong association between condom use and microbicide use as described in Trussell and Dominik [14]. Given the high efficacy of condoms, most of the HIV infections that are observed in a trial are from vaginal (or anal) intercourse unprotected by condoms. Therefore, a positive association between condom and gel use would lead to stronger dilution effects than those presented in this manuscript. Similarly, other behavioral factors which affect adherence might lead to stronger/weaker dilution effects. For instance, if gel users tend to be not very sexually active then stronger dilution effect might be expected since most of the HIV infections in the trial will happen among sexually active women.

Importantly, dilution effects could also reduce the effect of a (truly) harmful product which increases the risk of HIV infections. Thus, some of the candidate microbicides which have been show to increase the risk of HIV acquisition may have been more harmful than what was observed in these trials.

A case in point is the Carraguard trial which specifically discussed and attributed the lack of effectiveness to low adherence, HIV infections from AI, and the potential efficacy of the placebo gel used in the trial [2]. Based on a staining assay technique, they have estimated the adherence in the trial to be only 42% (although this estimate might be an under-estimate [15]). Assuming a true efficacy of 50% in our model, an overall expected effectiveness of only 14.9% is obtained with an adherence of 40%, 20% of HIV infections from AI, a placebo efficacy of 10%, and 0% time off-product (pregnant women were discontinued permanently from this trial). The observed effectiveness in the trial was 13% (95% CI [-9%:31%]). Given the potential amount of efficacy dilution in the trial, there was very little chance of detecting the effectiveness of the product if the true efficacy of Carraguard was in the range of 40%-60%. Furthermore, if the true efficacy was in the range of 75%, the overall expected effectiveness under our model would be only 25%, which remains much lower than the 33% the trial was powered to detect. Therefore, if Carraguard was truly moderately efficacious it is highly unlikely that this trial could have detected it. As a consequence of its observed effectiveness, Carraguard may well be discarded from the development pipeline. However, it is interesting to speculate whether it would meet the same fate had it been evaluated under more controlled sources of dilution.

Even though a moderately effective microbicide (30% to 50%) may not be sufficient to eradicate the HIV epidemic, it is nevertheless important to measure the extent of dilution to identify potentially efficacious products for further development, particularly as several moderately effective products with different mechanisms of action could be combined into a single very effective product (e.g., combining a barrier with an entry inhibitor).

Currently planned trials for new candidate microbicides have not fully resolved the problems related to dilution. To reduce the impact of time off-product due to pregnancy, some trials are now requiring that women use two methods of contraception before being allowed to enroll. Given the lack of safety data, it is not possible to allow women to remain on-product while pregnant. However, safety microbicide studies on pregnant women are currently on-going and more are being planned but it will take several years before the results become available from these studies.

Optimizing product adherence is essential. One key issue is the lack of a reliable assessment tool. So far, most of the assessment tools used in microbicide trials have relied on self-reported product use obtained via face-to-face interviews, which has been shown to produce over-estimation of adherence. Newly designed microbicide trials are planning to assess adherence using better assessment tools (e.g., audio computer-assisted self-interview) in order to increase the validity of the adherence data, which may also help to develop strategies to maximize and optimize adherence.

As with adherence, greater effort should be placed to use validated tools to collect data on AI. The dilution effect due to HIV infections that are not from vaginal intercourse is potentially large. Trial data cannot distinguish between anal and vaginal transmission. It is therefore important to improve estimates of the frequency of unprotected AI and to greatly intensify the counseling message to reduce the practice of unprotected AI during the trial. Ultimately, the best option would be to have a candidate product that can be used vaginally and anally as it would potentially provide the greatest benefit. Development of microbicides for AI is currently underway although most products are for vaginal use only.

A suggestion to control for all these different sources of dilutions is that the ITT analysis should not be the primary analysis in a microbicide trial and that so called 'per-protocol' analysis should be used where non-adherent women and time off-product are excluded from the analysis. We think that this is not a viable solution as it is problematic for two reasons. First, the benefit induced by randomization under an ITT analysis often outweighs what can be gained from a non-ITT analysis. For this reason, regulators (such as FDA) typically put a lot of weight on the ITT analysis. It is difficult to foresee how a candidate microbicide could be marketed solely based on its favorable results on a 'per-protocol' analysis with non-significant ITT results. Second, exclusion from the analysis of non-adherent women and/or of women engaging in unprotected AI needs to be done with a validated tool for assessing adherence and risk behaviors which has proven to be a great challenge so far.

Finally, a careful selection of the effect size to be detected in light of the potential dilution factors should be made in the planning of future trials. Protocol teams have used different effect size as it can be seen in past microbicide trial designs where efficacy varying from 30% to 50% have been used to determine the sample size.

## Competing interests

KD is an employee of Sanofi-Pasteur, Lyon, France. BRM, DD, and MCB declare that they have no competing interests.

## Authors' contributions

BRM derived the analytical formulas for the dilution and drafted the manuscript. MCB extracted the data from the published literature and lay out the different scenarios. DD carried out the simulations and produced the data that were used in the figures. KD participated in the analysis of the simulation data and helped to draft the manuscript. All authors read and approved the final manuscript.

## Supplementary Material

Additional file 1 **Results of completed effectiveness trials of candidate vaginal microbicides for the prevention of HIV acquisition **[1-3,16-20]. Table showing the results of completed effectiveness trials of candidate vaginal microbicides for the prevention of HIV acquisition. Click here for file

Additional file 2 **Description and computational details of the different dilution factors, expected effectiveness and total dilution effect **[9,21]. Table showing the description and computational details of the different dilution factors, expected effectiveness and total dilution effect. Click here for file

Additional file 3 **Percent reduction (*D*) and expected effectiveness (*E*_*all*_) for four scenarios of dilution with different true efficacy of active gel (*E*_*T*_)**. Table showing the percent reduction (*D*) and expected effectiveness (*E*_*all*_) for four scenarios of dilution with different true efficacy of active gel (*E*_*T*_). Click here for file
