# Supplementary material for: Efficacy dilution in randomized placebo-controlled vaginal microbicide trials
Source: Emerg Themes Epidemiol. 2009 Oct 9;6:5. doi: 10.1186/1742-7622-6-5 (PMC2768687; doi:10.1186/1742-7622-6-5)
Supplement: Additional file 1 — Results of completed effectiveness trials of candidate vaginal microbicides for the prevention of HIV acquisition [1-3,16-20]. Table showing the results of completed effectiveness trials of candidate vaginal microbicides for the prevention of HIV acquisition. [file 1742-7622-6-5-S1.DOC]

| **Clinical Trial** | **Design / Main Results** | | **Potential Sources of Efficacy Dilution** | | | |
| --- | --- | --- | --- | --- | --- | --- |
| **Product /**  **Formulation /**  **Mechanism /**  **Formulation /**  **Date of trial /**  **Main Reference /**  **Main Conclusion** | **Phase / Trial Population / Country** | **Sample Size /**  **Observed Number of HIV Endpoints / Hazard Ratio /**  **(95% CI)** | **Adherence &**  **Product Use** | **Time off product due to pregnancy** | **Anal Intercourse** | **Type of Placebo /**  **Efficacy of Placebo** |
| COL-1492  Nonoxynol-9 52.5mg  Vaginal Gel  Surfactant  1996-2000  Van Damme et al [16]  Multiple use of N-9 could cause toxic effects enhancing HIV-1 infection. | Phase II/III  -2 arms  -triple blind placebo controlled  -Condom promotion in both arms  -Female sex worker  - Benin, Cote d’Ivoire, South Africa, Thailand | 892 total  -449 on N-9  -443 on placebo  Observed HIV infections  -59 on N-9  -45 on placebo  HR: 1.5 (1.0-2.2) | Proportion of vaginal sex acts with clients using study gel:  -79% on N-9  -81% on placebo | Exclusion of women who wanted to become pregnant in the coming year  10 women were early terminated for pregnancy during the trial | 14% ever had anal sex at baseline  Median frequency of 2.0 per week  In Durban 75% of participants reported UAI during follow-up | A vaginal moisturiser marketed as Replens; same as COL-1492 but did not contain N-9 but had more carbomer which has in vitro activity against HIV  Unknown efficacy; possible barrier and lubrification effect |
| Nonoxynol-9 70mg  Vaginal Film  Surfactant  1994-1996  Roddy et al [17]  The use of a N-9 vaginal film did not reduce the rate of HIV infection. | Phase III  -2 arms  -double blind placebo controlled  -Condom promotion in both arms  -Female sex worker  -Cameron | 1292 total  - 648 on N-9  - 644 on placebo  Observed HIV infections  - 46 on N-9  - 48 on placebo  HR: 1.0 (0.7-1.5) | Proportion of vaginal sex acts with clients using study film:  -89% on N-9  -87% on placebo | Exclusion of women who wanted to become pregnant in the coming year  No data available on pregnancy observed during follow-up | Ever had anal sex at baseline:  - 5% in N-9  - 6% in placebo | Vaginal film without N-9  Unknown efficacy; possible barrier and lubrification effect |
| Savvy  C31G 1.0%  Vaginal Gel  Surfactant  2004-2006  Peterson et al [18]  Trial was stopped prematurely because of low HIV infection rates | Phase III  -2 arms  -double blind placebo controlled  -Condom promotion in both arms  -Sexually active women at risk of HIV infection  - Ghana | 2142 total  - 1073 on Savvy  - 1069 on placebo  Observed HIV infections  - 8 on Savvy  - 9 on placebo  HR: 0.9(0.3-2.3) | Mean percentage of vaginal sex acts with study gel:  -75% on Savvy  -77% on placebo | -Exclusion of women who wanted to become pregnant in the coming year  -Kaplan-Meier pregnancy probability at 12 mos:  -43% on Savvy  -44% on placebo  -10% of person-time off product during follow-up | Anal sex in previous 30 days at baseline:  -1.8% on Savvy  -2.5% on placebo | Isotonic with a 4.4 pH; contains hydorxyethy-cellulose as a gelling agent and sorbic acid  Unknown efficacy; placebo gel induced a decrease in semen pH; possible barrier and lubrification effect |
| Savvy  C31G 1.0%  Vaginal Gel  Surfactant  2004-2006  Feldblum et al [19]  Trial was stopped prematurely because of futility | Phase III  -2 arms  -double blind placebo controlled  -Condom promotion in both arms  -Sexually active women at risk of HIV infection  -Nigeria | 2153 total  - 1076 on Savvy  - 1077 on placebo  Observed HIV infections  - 21 on Savvy  - 12 on placebo  HR: 1.7(0.9-3.5) | Mean percentage of vaginal sex acts with study gel:  -78% on Savvy  -79% on placebo | -Exclusion of women who wanted to become pregnant in the coming year  -Proportion of participants who became pregnant at least once during follow-up:  -25% on Savvy  -26% on placebo  -5% of person-time off product during follow-up | Anal sex in previous 30 days at baseline:  -4% overall | Isotonic with a 4.4 pH; contains hydorxyethy-cellulose as a gelling agent and sorbic acid  Unknown efficacy; placebo gel induced a decrease in semen pH; possible barrier and lubrification effect |
| Carrageenan  Carraguard/PC515  Vaginal Gel  Entry inhibitors ; anionic polymers  2004-2007  Skoler-Karpoff et al [2]  Carraguard is safe; not effective in preventing HIV acquisition | Phase III  -2 arms  -double blind placebo controlled  -Condom promotion in both arms  -Sexually active women at risk of HIV infection  -South Africa | 6202 total  -3011 on Carraguard  -2994 on placebo  Observed HIV infections  - 134 on Carraguard  - 151 on placebo  HR: 0.9(0.7-1.1) | Self-reported gel use with vaginal sex acts:  -96% on Carraguard  -96% on placebo  Gel use with vaginal sex acts from staining assay:  -41% on Carraguard  -43% on placebo | Exclusion of women who wanted to become pregnant in the coming year.  Pregnant women were discontinued from the trial  11% in both arms became pregnant during follow-up | Unprotected anal sex in past 3 months at baseline:  -2% in both arms | Methyl-cellulose gel  Unknown efficacy; possible barrier and lubrification effect |
| Cellulose sulphate 6%  Ushercell  Vaginal Gel  Entry inhibitors ; anionic polymers  2005-2007  Van Damme et al [1]  Trial was stopped prematurely because un-favourable interim efficacy results; Cellulose Sulphate may have increased the risk of HIV acquisition. | Phase III  -2 arms  -double blind placebo controlled  -Condom promotion in both arms  -Sexually active women at risk of HIV infection  - South Africa, Uganda, Benin, India | 1398 total  - 706 on CS  - 692 on placebo  Observed HIV infections  - 25 on CS  - 16 on placebo  HR: 1.6(0.9-3.0) | Proportion of vaginal sex acts with study gel:  -87% on CS  -87% on placebo | Exclusion of women who wanted to be pregnant in the coming year.  -Pregnancy (incidence) rates in trial:  - 22% on CS  - 23% on placebo  - 68% of women interrupted product use due to a positive pregnancy test | Anal sex in past month at screening:  -4.2% on CS  -3.2% on placebo | Isotonic with a 4.4 pH (cellulose sulphate pH is 7.5); contains hydorxyethy-cellulose as a gelling agent and sorbic acid    Unknown efficacy; placebo gel induced a decrease in semen pH; possible barrier and lubrification effect |
| Cellulose sulphate 6%  Ushercell  Vaginal Gel  Entry inhibitors ; anionic polymers  2004-2007  Halpern et al [20]  Trial was stopped prematurely after the parallel trial concluded that CS might be increasing the risk of HIV acquisition. | Phase III, double blind  -2 arms  -double blind placebo controlled  -Condom promotion in both arms  -Sexually active women at risk of HIV infection  -Nigeria | 1644 total  -820 on CS  -824 on placebo  Observed HIV infections  - 10 on CS  - 13 on placebo  HR: 0.8(0.3-1.8) | Proportion of vaginal sex acts with study gel:  -81% overall | Exclusion of women who wanted to be pregnant in the coming year.  -Pregnancy (incidence) rates in trial:  - 29% on CS  - 28% on placebo  Proportion of time off product due to pregnancy:  -5.1% on CS  - 4.5% on placebo | Anal sex in past month at screening:  -4.1% overall | Isotonic with a 4.4 pH (cellulose sulphate pH is 7.5); contains hydorxyethy-cellulose as a gelling agent and sorbic acid  Unknown efficacy; placebo gel induced a decrease in semen pH; possible barrier and lubrification effect |
| HPTN 035  (2 products)  Carbopol 974P BufferGel  Vaginal gel  Vaginal milieu protectors ; acidifying agents  Naphthalene sulfonate PRO2000 0.5%  Vaginal gel  Entry inhibitors ; anionic polymers  2005-2008  Abdool Karim et al [3]  PRO 2000 0.5% had a 30% lower rate of HIV acquisition compared to placebo but not statistically significant. BufferGel did not alter the risk of HIV infection. | Phase II/IIIb  -4 arms  BufferGel, PRO2000  Placebo gel (double-blind placebo controlled)  and  no gel (open-label arm)  -Condom promotion in all arms  -Sexually active women at risk of HIV infection  - Malawi, South Africa, Zambia, Zimbabwe, USA | 3099 total  -775 on Buffergel  -769 on PRO2000  -771 on placebo  -772 on ‘no gel’  Observed HIV infections  -54 on Buffergel  -36 on PRO2000  -51 on placebo  -53 on ‘no gel’  BufferGel vs placebo  HR: 1.1(0.8-1.6)  BufferGel vs ‘no gel’  HR: 1.1(0.7-1.6)  Pro2000 vs placebo  HR: 0.7(0.5-1.1)  PRO2000 vs ‘no gel’  HR: 0.7(0.4-1.0) | Mean percentage of vaginal sex acts with study gel:  -81% across gel arms | Exclusion of women who wanted to become pregnant in the coming years  -610 pregnancies during the trial  -Pregnancy (incidence) rates in trial:  rate 11.3%  -5.9% of person-time off product during follow-up in gel arms | 5% ever had anal sex at baseline | Placebo gel: Isotonic with a 4.4 pH; contains hydorxyethy-cellulose as a gelling agent and sorbic acid  Unknown efficacy; placebo gel induced a decrease in semen pH; possible barrier and lubrification effect  ‘No gel’: no biological effect but potential biases associated with an open label arm |
| MDP-301  (1 product : 2 doses)  Naphthalene sulfonate PRO2000 in 0.5% and 2% formulation  Vaginal gel  Entry inhibitors ; anionic polymers  2005-2009 [results expected at the end of 2009]  The 2% dose arm was halted in Feb. 2008 after it was found that it was statistically unlikely that the 2% formulation would show an effect against HIV acquisition. | Phase III  -3 arms  -PRO 2000 0.5%  -PRO 2000 2%  -Placebo gel double-blind placebo controlled  -Condom promotion in all arms  -Sexually active women at risk of HIV infection  - South Africa, Zambia, Tanzania, Uganda | - 9395 women followed for 1 year  -6600 women allocated to PRO 2000 0.5% and placebo gel | [study on-going] | -Exclusion of women who wanted to become pregnant in the coming year.  -Pregnant women are discontinued from the trial but will be censored at time of pregnancy in the primary analysis | [study on-going] | Placebo gel: Isotonic with a 4.4 pH; contains hydorxyethy-cellulose as a gelling agent and sorbic acid  Unknown efficacy; placebo gel induced a decrease in semen pH; possible barrier and lubrification effect |
