# Supplementary material for: Efficacy dilution in randomized placebo-controlled vaginal microbicide trials
Source: Emerg Themes Epidemiol. 2009 Oct 9;6:5. doi: 10.1186/1742-7622-6-5 (PMC2768687; doi:10.1186/1742-7622-6-5)
Supplement: Additional file 2 — Description and computational details of the different dilution factors, expected effectiveness and total dilution effect [9,21]. Table showing the description and computational details of the different dilution factors, expected effectiveness and total dilution effect. [file 1742-7622-6-5-S2.DOC]

| **Sources of Dilution** | | **Description** | **Expected Effectiveness** | **Assumptions/Notes** |
| --- | --- | --- | --- | --- |
| **Parameter** | |  |  |  |
|  | *E_T_* | True efficacy of a candidate microbicide gel compared to an inert product (i.e. a “no-gel” control group). | *E_T_* | **Assumption:** Optimal and maximum efficacy of a candidate vaginal gel microbicide in absence of any source of bias or dilution. This assumption removes all the potential biases that could be associated with the open-label nature of a comparison with a “no gel” control group. |
| **Adherence/Gel Use** | *a* | The proportion of women in the trial using the product as prescribed by the protocol for each vaginal intercourse during the entire follow-up. | *E_a_ = a * E_T_* | **Assumption**: Adherence is not associated to the treatment allocation (i.e. no differential adherence by study arm) and to any risk factors for HIV.  **Note:** The formula for *E_a_* is less conservative than the usual formula for adjusting sample size for adherence which assumes that non-adherent women in the placebo arm have a HIV acquisition rate similar to those in the active arm (cross-over assumption) [21]. Since non-adherent women do not benefit from the product, the cross-over assumption is hard to justify in the context of vaginal microbicide trials. |
| **Time Off-Product due to Pregnancy** | *o* | The proportion of total follow-up time off-product due to pregnancy (i.e. *o* equals the follow-up time off-product divided by the total follow-up time in the trial). | *E_o_= (1-o)*E_T_* | **Assumption:** The candidate microbicide gel is not contraceptive. In addition, women off-product have the same HIV acquisition rate as women in the placebo arm.  **Notes:** Some of the products in evaluation might be contraceptive. In addition, some studies have shown an increase in the HIV acquisition rate during pregnancy. This could further complicate the interpretation of trial’s results since the biases induced by the contraceptive effect and the change in HIV acquisition rate during pregnancy could go in either direction but it would be extremely difficult to determine the direction of the bias using the data from the trial [9]. |
| **Source of Infection: Anal Intercourse (AI)** | *s* | The proportion of HIV infections obtained at the end of trial that have been acquired from unprotected receptive AI. | *E_s_ = 2E_T *_ (1-s)/(2- sE_T_)* | **Assumption:** The candidate microbicide gel is not used during AI and is ineffective against the acquisition of HIV during unprotected receptive AI.  **Note:** The formula for *E_s_* can be derived as follow: a total of *N* HIV infections is observed at the end of the trial where *sN* and *(1-s)N* HIV infections have been acquired from unprotected receptive AI and vaginal intercourse, respectively. On average, the *sN* HIV infections will be equally split between the active and placebo arms with *sN/2* HIV infections in each arm. Given the efficacy *E_T_*, the *(1-s)N* remaining HIV infections will divide into *(1-E_T_)(1-s)N/(2-E_T_)* and *(1-s)N/(2-E_T_)* between the active and placebo arms, respectively. Finally, *(1-E_s_)* is obtained by dividing the total number of HIV infections in the active arm by the one observed in the placebo arm. |
| **Placebo Physical Barrier /Lubrication Effect** | *E_p_* | True efficacy of the vaginal placebo gel | *E_b_=1– ((1-E_T_)/(1-E_p_))* | **Assumption:** When *E_T_ < E_p_* (i.e. the placebo gel is more efficacious than the active gel), *E_b_* will be negative (< 0%). In this paper we are only considering *E_T_ > E_p_* (i.e. the efficacy of the placebo gel is smaller than the one for the candidate microbicide gel).  **Note:** The formula for *E_b_* can be derived as follow: *(1-E_p_)* is defined as the ratio of the HIV acquisition rate observed in the placebo gel arm (*I_p_*) and the one in the inert control arm (*I_c_)*, thus *(1-E_p_) = I_p_ / I_c_*. If *I_a_* is the HIV acquisition rate observed in the active gel arm then *(1-E_b_) = I_a_ / I_p_ = I_a_ / ((1-E_p_) * I_c_ ) = (1-E_T_) / (1-E_p_)*. |
| **Combined Dilution Factors** | *E_all_* | The expected effectiveness in a trial taken into account all the above sources of dilution. | *E_all_= 2a(1-s)(1-o)(E_T_-E_p_) / ((2(1-E_p_)) – (s(E_T_-E_p_)))* | **Assumption:** The following natural ordering of dilution effects is used:  *E_T_ → E_b_ → E_s_ → E_o_ → E_a_* *→ E_all_*  First, we start by taking into account the efficacy of the placebo gel followed by the dilution effect due to HIV infections from AI. For the two remaining source of dilutions, adherence and time off- product, the order is not important since the dilution effects for both are linear therefore; the ordering does not alter the expression for *E_all_*.  **Note:** The above formulation implies that all four sources of dilution are independent from each others. This is a reasonable assumption for the dilution due to the placebo gel efficacy but one can argue that frequency of AI, risk of pregnancy, and adherence might be associated. A strong positive association between these dilution sources could reduce the total dilution effect. However, strong associations between these factors in microbicide trials have not been observed to date. More complex models could be used which could include associations between the different sources of dilution. Those models would require more complex assumptions on the nature and magnitude of these associations as well as the availability of validated tools to evaluate these associations in trials. |
| **Total Dilution Effect** | *D* | *D* is the percent reduction from *E_T_* to *E_all_* | *D = 1 – (E_all_ /E_T_)* |  |
