# Supplementary material for: Efficacy dilution in randomized placebo-controlled vaginal microbicide trials
Source: Emerg Themes Epidemiol. 2009 Oct 9;6:5. doi: 10.1186/1742-7622-6-5 (PMC2768687; doi:10.1186/1742-7622-6-5)
Supplement: Additional file 3 — Percent reduction (D) and expected effectiveness (Eall) for four scenarios of dilution with different true efficacy of active gel (ET). Table showing the percent reduction (D) and expected effectiveness (Eall) for four scenarios of dilution with different true efficacy of active gel (ET). [file 1742-7622-6-5-S3.DOC]

| **Source of Dilution** | **Parameter** | **Scenario** | | | |
| --- | --- | --- | --- | --- | --- |
|  |  | **1** | **2** | **3** | **4** |
| Adherence/Product Use | *a* | **90%** | **80%** | **70%** | **60%** |
| Time off product due to pregnancy and/or adverse event | *o* | **5%** | **10%** | **15%** | **20%** |
| Source of HIV infection: Anal Intercourse | *s* | **5%** | **10%** | **20%** | **30%** |
| Placebo gel efficacy | *E****_p_*** | **0%** | **0%** | **5%** | **10%** |
| **Results** | **True Efficacy**  **(*E_T_*)** | **Total dilution effect (*D*)**  **[Expected Effectiveness *E_all_*]** | | | |
|  | *E_T_* = 75%  *E_T_* = 50%  *E_T_* = 25% | **17.2% [62.1%]**  **17.8% [41.1%]**  **18.3% [20.4%]** | **32.7% [50.1%]**  **33.5% [33.2%]**  **34.4%**  **[16.4%]** | **49.2% [38.1%]**  **52.4% [23.8%]**  **58.8% [10.3%]** | **63.0%**  **[27.7%]**  **67.4% [16.3%]**  **76.6% [5.8%]** |
